# Developing a post-stroke home care checklist for primary care professionals in Turkey: a modified Delphi study

**DOI:** 10.1017/S146342362300004X

**Published:** 2023-03-27

**Authors:** Esra Akgül, Serap Çifçili, Çiğdem Apaydın Kaya

**Affiliations:** 1 Department of Family Medicine, Marmara University School of Medicine, Istanbul, Turkey; 2 Eastern Mediterranean University School of Medicine, Istanbul, Turkey

**Keywords:** checklist, Delphi technique, elderly, home care, primary care, stroke

## Abstract

**Aim:**

The aim of this study is to develop a post-stroke home care checklist for the use of primary care professionals.

**Background:**

Home care is an integral part of primary health care. In the literature, several scales are available to help determine elderly individuals’ need for home care services; however, there are no standard care criteria or guidelines for the home care of stroke survivors. Therefore, a standardized post-stroke home care tool specific for use by primary care professionals is needed to identify patients’ needs and to detect intervention areas.

**Methods:**

This is a checklist development study carried out between December 2017 and September 2018 in Turkey. A modified Delphi technique was used. In the first stage of the study, a literature review was carried out, a workshop was conducted with healthcare specialists in the stroke area, and a 102-item draft checklist was created. In the second stage, two written Delphi rounds were carried out via email with 16 healthcare professionals providing post-stroke home care. In stage three, the agreed items were reviewed, and similar items were grouped together to create the final checklist.

**Findings:**

A consensus was achieved in 93 of the 102 items. The final checklist, consisting of four main themes and 15 headings, was created. The four main areas of assessment in post-stroke home care are ‘assessment of current status’, ‘identification of risks’, ‘evaluation of the care environment and caregiver’, and ‘planning follow-up care’. The Cronbach alpha reliability coefficient of the checklist was found to be 0.93. In conclusion, the PSHCC-PCP is the first checklist created to be used by primary care professionals in post-stroke home care. However, it needs to be assessed in terms of effectiveness and usefulness with further studies.

## Introduction

Stroke is the second leading cause of death and third leading cause of disability worldwide (World Health Organization, 2018). Continuity of care for stroke survivors following discharge from hospital should be provided in the community by primary care professionals (Venketasubramanian *et al.*, 2008). Providing health services to individuals at home or in the environment where they live, in the context of family, community, and culture is among the practices of primary care professionals. In other words, home care is an integral part of primary health care (Freeman, 2016). In Turkey, primary health care is served in the Family Health Centers by the professionals consisting of family physicians, and nurses or midwifes. Therefore, post-stroke home care is also a part of the duty of those primary care professionals. Family physicians provide home care to their registered homebound patients. In addition to the Family Health Centers, home health services are also provided in the home care units affiliated to the public or private hospitals. In these units, home care is provided by family physicians and nurses.

In the literature, several scales are available to help determine elderly individuals’ need for home care services (Gokler *et al.*, 2015); however, there are no standard care criteria or guidelines for the home care of stroke survivors. Therefore, a checklist is needed to help improve the quality of health care provided to stroke survivors by primary care professionals. Different national healthcare systems use various post-stroke care guidelines (National Institute for Health and Clinical Excellence, 2008; Uzuner, et al, 2015; Iosa *et al.*, 2018). However, either they were developed for hospital care or do not fully address the needs of patients (Venketasubramanian *et al.*, 2011; Philps *et al.*, 2013). The Global Stroke Community Advisory Panel developed a post-stroke checklist that was found to be useful (Ward *et al.*, 2014), but primary care professionals were not represented on the expert panel who developed the checklist (Turner *et al.*, 2019). A standardized post-stroke home care tool specific for use by primary care professionals is needed to identify patients’ needs and to detect intervention areas. The aim of this study was to develop a checklist for post-stroke home care for the use of primary healthcare professionals.

## Materials and methods

This is a survey development study using the modified Delphi technique (Boulkedid *et al.*, 2011 and Custer *et al.*, 1999), which was carried out between December 2017 and September 2018, in Istanbul, Turkey. The aim of using the Delphi technique is ‘to achieve agreement among a group of experts on a certain issue where none previously existed’ (Boulkedid *et al.*, 2011). The technique is characterized by a set of structured communication methods to facilitate consensus of opinion among experts on a prespecified content area through a series of questionnaires combined with controlled feedback. During each round of activity, information is collected from experts anonymously by a Delphi moderator and returned to the panelists for comment (Dalkey and Helmer, 1963). Each round summarizes information presented in the previous round, which is then presented again to experts for prioritization to establish group agreement. Different types of Delphi methods have emerged over time from the original Delphi due to the flexible nature of the technique (Hsu and Sandford, 2007). In the modified Delphi technique, the first round can be performed using different types of studies, such as literature review or expert group panel session.

In this study, for the first stage of the modified Delphi technique, we reviewed literature to reach systematic reviews and national and international guidelines and listed the problems and recommended solutions related to post-stroke home care. Then, a workshop was conducted with healthcare professionals to discuss the problems and intervention areas in post-stroke home care and the needs of patients and caregivers.

For the second stage of the Delphi technique, health professionals who are taking care of stroke patients and have at least one year of experience in this area were invited to be part of the expert panel for the written rounds by emailing. Those who accepted the invitation were included. The professionals who did not complete the second written round of the Delphi were excluded from the study.

Below is a detailed description of how each round was realized.

## Stage 1: Constructing a draft checklist

### Literature search

First, a literature search was conducted to determine the issues to be discussed in the workshop. A systematic search of the literature was conducted from PubMed and MEDLINE databases between 1 January 2007 and 31 December 2017. We used a combination of keywords, ‘home care’, ‘post stroke care’, ‘post stroke home care’, ‘stroke transitional care’. Systematic reviews and national and international guidelines focusing on post-stroke home care problems and solutions were included (Philps *et al.*, 2013; Uzuner *et al.*, 2015; Kirchhof *et al.*, 2016; Winstein *et al.*, 2016). Problems and recommended solutions identified in the relevant literature were listed by the researchers and presented for discussion by the expert members of the workshop.

### Workshop

We invited the professionals who have been working with post-stroke patients (academic staff of neurology, physical medicine and rehabilitation and family medicine departments) and family physicians providing home care in family health centers, by mailing to Marmara University School of Medicine Hospital and family health centers’ mail groups. Those who accepted this invitation were included in the workshop. The workshop members consisted of seven healthcare professionals: one neurologist, one physical medicine and rehabilitation physician, three family medicine academicians, and two family physicians, currently working at the family health centers. In the beginning of the workshop, the purpose of this study was explained to the participants by the researchers. Afterward, the following questions were asked of the workshop members:

- What problems do you encounter while providing home care for post-stroke patients?
- What items would you recommend be included in a post-stroke home care checklist to be prepared for family physicians or primary care professionals?
- What scales should be used to evaluate stroke survivors’ health condition and/or treatment follow-up at home?

Additionally, the problems and intervention areas related to post-stroke care and recommendations gathered from the relevant literature were discussed in the workshop. The whole workshop session was recorded with a voice recorder. After analysis of the recording, a 102-item post-stroke home care checklist was drafted.

## Stage 2: Written Delphi rounds

After the draft checklist was created, in the Delphi’s second stage, written rounds were performed.

In Delphi exercises, a minimum of 12 respondents is generally considered to be sufficient to enable consensus to be achieved (Murphy *et al.*, 1998). Estimating a 50% response rate, 30 experts were invited to participate in the study by emailing. This invitation mail included detailed information about the purpose and method of the study and instructions about how to participate in the study was provided. To ensure the maximum variability, we purposively selected experienced participants from different professions who have been providing home care to post-stroke patients for at least one year. All the experts were clinicians who were actively involved in providing post-stroke home care facilities. Sixteen of the invited experts completed all the Delphi rounds (53% of the invitees). These experts were specialists in family medicine (*n* = 5), neurologists (*n* = 3), physical medicine and rehabilitation physicians (*n* = 2), nurses (*n* = 2), general practitioners (*n* = 2), a geriatrician (*n* = 1), and a social worker (*n* = 1). Three of the specialists in family medicine and both of the two general practitioners were actively working in family health centers.

The experts who participated in the first stage (the workshop) were not invited to the second stage (written Delphi rounds), and the professionals on the expert panel were unaware of the identity of the other panelists. Since a consensus was achieved in 91% of the items by the second round, a third round was not conducted.

### First round

In the first round, we asked the experts to evaluate the draft 102-item, post-stroke home care checklist via email. The experts were asked to rate the importance of the items for the checklist according to whether they were ‘useful’ and ‘simple and practical’, whether they ‘focused on the main problematic areas after stroke’, and whether they constituted ‘an evidence-based intervention that contributes to the patient’s quality of life’ on a 5-point Likert-type scale from ‘not important at all’ to ‘very important’. In addition, there was an ‘I have no idea’ choice and a section for providing additional comments about each item.

### Second round

In the second round, we sent the same checklist to the same experts again via email, having added a statistical analysis of the answers given in the first round. In the statistical analysis, the number of responders and the mean, median, and standard deviation of the rating points were calculated for each item. In addition, each respondent’s previous evaluation and opinions were added to the checklist. The experts were asked to review their responses again in the light of the opinions and responses of the other experts.

All the experts were reminded once a week via email to complete the Delphi evaluations. The first checklist items were submitted in March 2018 and the last one in June 2018.

## Stage 3: Constructing the final checklist

The agreed-upon items were reviewed according to the current literature, and then, similar items were grouped together to create the final checklist.

## Analysis

Descriptive statistics were used to describe participants’ responses to each item in the two written rounds. All ‘no idea’ responses were excluded from the group response to ensure that the reported percentage of agreement or disagreement for each statement represented the consensus among only those who considered themselves to have expertise related to the item.

In the second stage of the Delphi, to evaluate whether or not a consensus was achieved for each item, four statistical values were calculated: first quartile (Q1), median (M), third quartile (Q3), and range (R).

Consensus was based on two criteria: the median value of the experts’ ratings should be >3 of the median, and the difference between the interquartile ranges should be lower than 1.2. Items that met these criteria were included in the checklist (Zeliff and Heldenbrand, 1993). Lastly, we calculated the Cronbach alpha value to evaluate inter-item reliability. All analyses were conducted using SPSS for windows, version 20.0.

## Results

During the first stage, a draft checklist consisting of 102 items was created. Consensus was achieved for 86 items by the end of the first round. By the end of the second round, consensus had been achieved for an additional 11 items. However, for four items that had achieved consensus in the first round, consensus disappeared in the second round. As a result, consensus was finally achieved in 93 of the 102 items. No new items were added to the checklist by the Delphi experts. The items for which no consensus was achieved are presented in Table 1.

**Table 1.** Items for which no consensus was achieved

Items that were agreed on in the first round but for which agreement disappeared in round 2	First Round				Second Round			
	Q1	Med	Q3	R1	Q1	Med	Q3	R2
1. Assessment of dependency with ICF^*^	3	4	4	1	2.5	4	4	1.5
2. Assessment of the general hygiene of the patient	4	4	5	1	3.75	4	5	1.25
3. Assessment of the need for occupational therapy	3	4	4	1	3	4	4.25	1.25
4. Assessment of the suitability of the physical conditions of the home for the patient	4	4	5	1	3.75	4.5	5	1.25
Items not agreed on in both rounds								
1. Assessment of cognitive function using the Mini-Mental State Examination	2.5	3	4	1.5	2	3	4	2
2. Assessment of dependency with the Barthel Index	3.75	4	5	1.25	3	4	5	2
3. Assessment of the patient in terms of sexual dysfunction	2	3	4	2	2	3	4	2
4. Assessment of the caregiver burden	3.75	4	5	1.25	3.75	4	5	1.25
5. Questioning the presence of more than one caregiver and reviewing the follow-up plan if necessary	3	4	4.25	1.25	3	4	5	2

* : The International Classification of Functioning, Disability, and Health.

Q1 (First Quarter): The point where 25% of the answers are listed on the left, and 75% of the answers are listed on the right.

Med (Median): The point where 50% of the answers are listed on the left, and 50% of the answers are listed on the right.

Q3 (Third Quarter): The point where 75% of the answers are listed on the left, and 25% of the answers are listed on the right.

R (Range): The difference between the third quarter and the first quarter (R = Ç3−Ç1). According to Zeliff *et al.*, this difference being lower than 1.2 indicates that there is a consensus, whereas it being higher indicates that there is no consensus (18).

R1: Range in the first round.

R2: Range in the second round.

**Figure 1.**
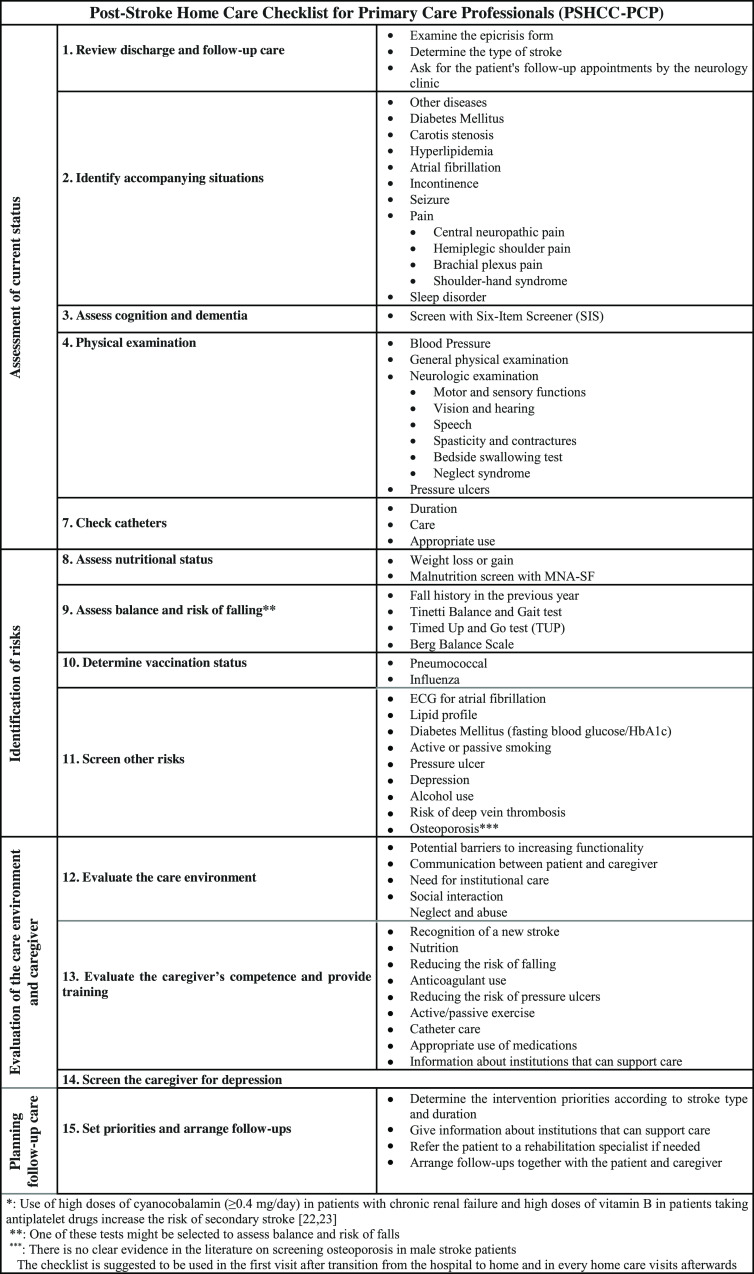
Post-stroke home care checklist for primary care professionals (PSHCC-PCP)

The items that the experts agreed on were placed into four main groups under 15 headings according to their commonalities (Figure 1). The four main groups are ‘assessment of current status’, ‘identification of risks’, ‘evaluation of the care environment and caregiver’, and ‘planning follow-up care’.

The Cronbach alpha reliability coefficient of the checklist was found to be 0.93.

Although a consensus was achieved on the item ‘assess calcium and vitamin D supplementation needs’, it was, nonetheless, changed to ‘assess vitamin D supplementation need’. This is because, in the relevant current literature, it was found that calcium supplements taken alone increased the risk of cardiovascular diseases (Li *et al.*, 2012), and high doses of calcium (≥1,000 mg/day) taken alone increased the risk of stroke; however, this risk was not observed when calcium was taken in combination with vitamin D (de Abajo *et al.*, 2017).

A consensus was reached on the assessment of balance and the risk of falling. Moreover, the panel experts concluded that it was important to use all the assessments included in the draft checklist to assess balance and the risk of fall; these included ‘falling history in the past year’, Itaki Fall Risk Scale, Tinetti Balance and Walk Test, Timed ‘Up and Go’ Test, and Berg Balance Scale. However, Itaki Fall Risk Scale was removed from the checklist as its use is recommended in a hospital setting (General Directorate of Health Services, Department of Quality and Accreditation in Health, 2016).

Some explanations and warnings were added at the end of the checklist by the researchers according to the literature, for example, ‘high doses of cyanocobalamin (≥0.4 mg/day) in patients with chronic renal failure or high doses of vitamin B in patients using antiplatelet treatment increase the risk of secondary stroke’ (Arshi *et al.*, 2015; Hankey, 2018). In addition, information about the evaluation and use of the scales that are recommended was added.

## Discussion

In this study, which aimed to develop a checklist to enhance post-stroke care in home care visits by primary care professionals, a checklist encompassing four main themes and 15 headings was developed using the modified Delphi method. The main themes in the finalized post-stroke home care checklist are ‘assessment of current status’, ‘identification of risks’, ‘evaluation of the care environment and caregiver, and ‘planning follow-up care’ (Figure 1).

In another post-stroke checklist study similar to the present study, Philps *et al.* (2013) created a checklist that consists of 11 long-term problem areas experienced by stroke survivors. This checklist provides example language that can be used to ask about the specified post-stroke problem areas and links patient responses to specific referrals. This checklist was reported to be created for use in primary care settings; however, the Delphi panel experts in this study consisted of various specialties but did not include any primary healthcare professionals. In our study, primary care professionals and a social worker were included in the Delphi panel in addition to the experts reported in the study by Philps *et al.* No standard is specified for the number and variety of Delphi panel experts (Hsu and Sandford, 2007 ). Therefore, subsequent studies are needed to determine the effectiveness and usefulness of tools produced using the Delphi technique. Another difference between the checklist developed by Philps *et al.* and the checklist developed by the present study is that the latter was specifically created for use in the context of home care of post-stroke patients, considering the problems and intervention areas that are specific to home care; therefore, the needs of caregivers, that stated as an important issue in the literature, are included in the checklist developed by the present study (Graven *et al.*, 2013; Scholten *et al.*, 2019).

The Delphi method does not specify any criterion for selecting Delphi participants (Hsu and Sandford, 2007). It is recommended that participants have backgrounds and experience related to the target issue. We invited 30 experts, 16 of whom completed the study (53%). These experts had various specialties, and they were all experienced in the home care of post-stroke patients (ie neurologist, physical medicine and rehabilitation physicians, geriatrician, family medicine specialist, general practitioner, nurse, and social worker).

Because experts have argued over the years that the median should be used as the measure of central tendency for Likert-scale data (Jamieson *et al.*, 2004; Hsu and Sandford, 2007), median scores and interquartile ranges were used to identify consensus in the present study. Different consensus criteria have been reported, for example, mean, mode, and consistency in standard deviations between the tours (Hsu and Sandford, 2007). Additionally, in this study, since a consensus was achieved in 91% of the items by the second round, a third round was not conducted. The results of a systematic search in which all Delphi studies (including modified Delphi) published between 1978 and 2009 were reviewed indicated that there is no clear scientific evidence about the optimal number of rounds; however, the recommended number of rounds is two or three (Boulkedid *et al.*, 2011).

Although ‘general physical examination’ was among the items in the draft checklist in our study, the consensus of the experts was to add ‘blood pressure measurement’ as a separate item; this shows the importance of blood pressure measurement in stroke follow-ups.

The strengths of this checklist are that it was created by experts in various fields especially for use by primary care professionals providing post-stroke home care and includes the needs of patients and caregivers. Another strength of this study is that the modified Delphi technique was used; the use of a modified Delphi process is appropriate if basic information concerning the target issue is available and usable (as in the subject of stroke care) (Hsu and Sandford, 2007). Moreover, the use of the Delphi technique in the development of the checklist provided participants with anonymity, which could reduce the effects of dominant individuals often experienced during group-based processes (Hsu and Sandford, 2007). This study also has some limitations. Although the Delphi method does not specify any criterion for selecting Delphi participants, occupational therapist could be included in the group of experts. However, there are limited numbers of occupational therapist in Turkey. Physiotherapists working in cooperation with physical medicine and rehabilitation physicians cover this area. Including the participants from various disciplines to Delphi Panel also might be a barrier to reach consensus on certain items, especially on the use of the indexes included in the checklist.

The PSHCC-PCP was developed for use in home care settings. The checklist is suggested to be used in the first visit after transition from hospital to home and in every home care visits afterward. In conclusion, the PSHCC-PCP is the first checklist created to be used by primary care professionals in post-stroke home care. However, effectiveness and usefulness of the checklist needs to be verified by real practice.
