# Developing a post-stroke home care checklist for primary care professionals in Turkey: a modified Delphi study – CORRIGENDUM

**DOI:** 10.1017/S146342362300052X

**Published:** 2023-12-04

**Authors:** Esra Akgül, Serap Çifçili, Çiğdem Apaydın Kaya

The authors of the above article regret that an incorrect version of Figure 1 was supplied. The correct version of the figure is placed on the next page.

**Figure 1.**
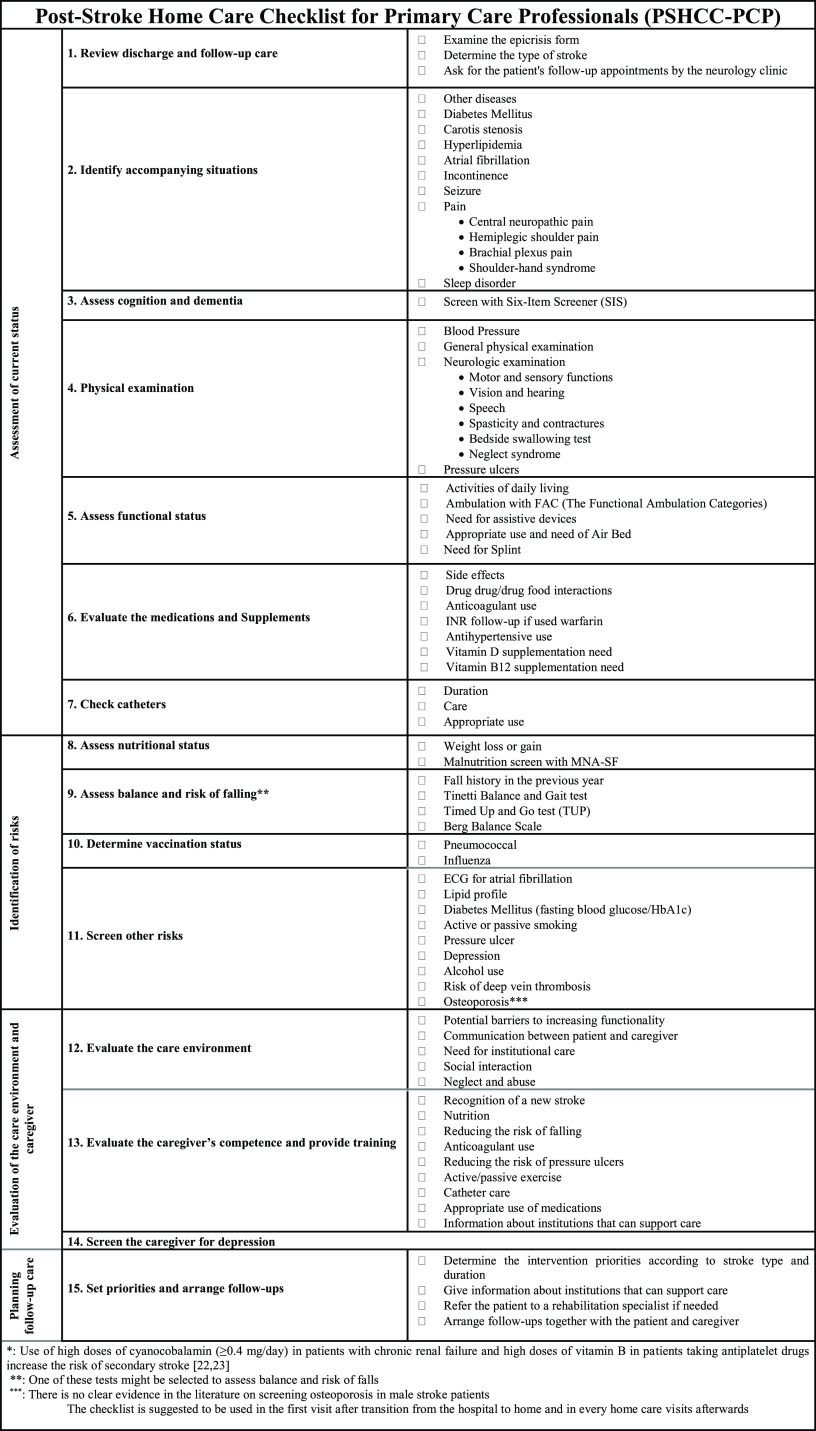
Post-stroke home care checklist for primary care professionals (PSHCC-PCP).
